# The dual interaction of antimicrobial peptides on bacteria and cancer cells; mechanism of action and therapeutic strategies of nanostructures

**DOI:** 10.1186/s12934-022-01848-8

**Published:** 2022-06-18

**Authors:** Atefeh Parchebafi, Farzaneh Tamanaee, Hassan Ehteram, Ejaz Ahmad, Hossein Nikzad, Hamed Haddad Kashani

**Affiliations:** 1 Anatomical Sciences Research Center, Institute for Basic Sciences, Kashan University of Medical Sciences, Kashan, Iran; 2 Department of Pathology, School of Medicine, Kashan University of Medical Sciences, Kashan, Iran; 3 Department of Pathology, Michigan Medicine, University of Michigan, Ann Arbor, MI USA

**Keywords:** Antimicrobial peptide, Cancer, Drug delivery, Anticancer peptide, Bacteria, Bacitracin

## Abstract

Microbial infection and cancer are two leading causes of global mortality. Discovering and developing new therapeutics with better specificity having minimal side-effects and no drug resistance are of an immense need. In this regard, cationic antimicrobial peptides (AMP) with dual antimicrobial and anticancer activities are the ultimate choice. For better efficacy and improved stability, the AMPs available for treatment still required to be modified. There are several strategies in which AMPs can be enhanced through, for instance, nano-carrier application with high selectivity and specificity enables researchers to estimate the rate of drug delivery to a particular tissue. In this review we present the biology and modes of action of AMPs for both anticancer and antimicrobial activities as well as some modification strategies to improve the efficacy and selectivity of these AMPs.

**Graphical Abstract:**

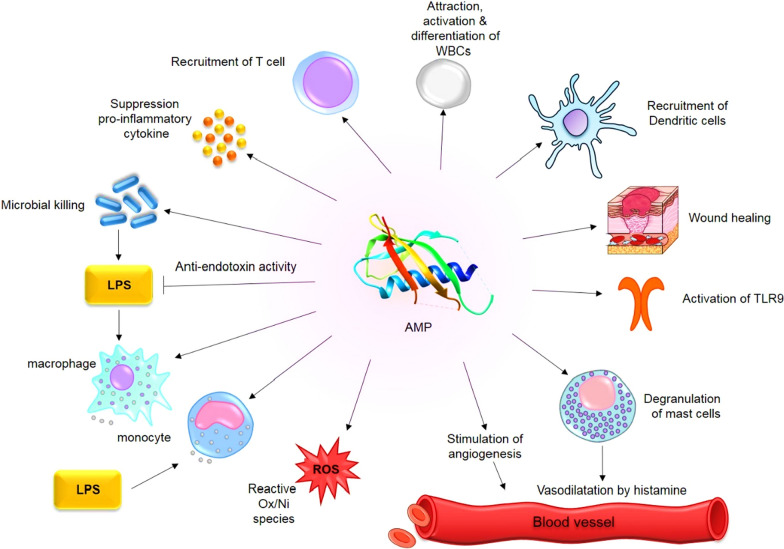

## Introduction

AMPs, also considered as host defense present in almost any living being from bacteria to plants and more complex ones such as vertebrates and invertebrates [1]. These hydrophobic amphipathic peptides are usually 10 to 50 residues long and demonstrate a net positive charge ranging from + 2 to + 13 [1, 2]. As a key component of innate immune system, they play a crucial role against resistant pathogenic organisms, making them a potential candidate for future antibiotic classes [3, 4]. Furthermore, these peptides have also proved to function as anticancer agents (ACP/Anticancer peptides) [5] with higher selectivity resulting in less side-effects than contemporary chemotherapeutics. Therefore, these peptides are novel candidates -for cancer therapy due to low toxicity (less side-effect), short time-frame of interaction (decreasing resistance probability), higher specificity, adequate solubility as well as tumor penetration [6, 7]. Although there are millions of natural and synthetic peptides known to us, but only a few of them have undergone clinical trials, mainly because of various challenges of these peptides as pharmaceutical drugs, for instance,their high synthesis cost is significantly problematic [7]. In this review we are focusing on these challenges and some modification strategies to improve these AMPs.

## What are the AMPs?

AMPs are cationic, amphipathic host defense peptides with a short length of 10 to 50 residues [8]. AMP genes have remained unchanged throughout natural selection and practically all living creatures from single-celled bacteria to those with multicellular organisms like plants, invertebrates and vertebrates beings tend to generate them. While AMPs in bacteria have the role of destroying other bacteria threatening their ecological niche, in more complex creatures, they are a crucial part of natural immunity and lead to defending the host against pathogens [9]. There are two groups of AMPs existing in bacteria functioning as bacteriocins: non-lantibiotics and lantibiotics. In 1947, the first bacterial lantibiotic AMP nisin was isolated from *Lactococcus lactis *[10]. AMPs affect not only a wide range of bacteria but also fungi, viruses and unicellular protozoa [11]. Many plants also contain AMP encoding genes leading to production of AMPs full of cysteine and disulfide bonds and ultimately use them as their main defense mechanism against microbial infections [12]. Thionins [13], plant defensins [14] and cyclotides are the best known examples of plant AMPs [15] which usually accumulate in leaves, flowers, seeds and tubers [16]. Just like plants, all invertebrates studied up-to-date do not benefit from an adaptive immune system and thus have to fully rely on innate immune system as their mechanism of defense [17]. A wide variety of AMPs have been found in vertebrates. For instance, the neutrophil granules in mammals contain AMPs and they are also secreted by epithelial cells. Up to date, over 500 AMPs have been found in amphibian skin glands [16]. Cathelicidin and defensins are two of the most significant AMPs in vertebrates [18].

### Types of AMPs

AMPs are classified based on their characteristics such as structure, sequence or mechanism of action like killing bacteria, immune modulation, preventing biofilm formation, and anti-cancer or anti-viral function [1].

AMPs classified by their secondary structures comprise α-helix, β sheet and extended/ random coil peptides [19, 20]. In aqueous solution, usually α-helix AMPs are unstructured, however, they show the amphipathic helical formation as they come in contact with trifluoroethanol, detergents/surfactants above critical micellar concentration such as sodium dodecyl sulfate (SDS) micelles and liposomes [21]. The two best known members of this category are (i) LL-37 [20] produced in neutrophils and epithelial cells as an inactive precursor in the 18 kDa human cathelicidin antimicrobial protein (hCAP18) [22], and (ii) human lactoferricin which can be found in milk and exocrine secretions and is derived by proteolytic cleavage of the antimicrobial and immunomodulatory iron binding glycoprotein lactoferrin [23]. To improve the anti-microbial activity in helix peptides, C-terminus amidation must be applied (Tables 1). This method also stabilizes the peptide localization at the cell's surface by increasing the electrostatic interaction between the cationic AMPs and the bacterial anionic membrane [24].

**Table 1:** Types of AMPs based on structure

Category	Peptides	Sequence feature	Source	References
α Helical peptides	Aurein 1-2 Mellitin Brevinin 1 Maculatins Citropin Buforin II Cathelicidins: -LL-37 -BMAP27,28,34 -Magainins Cecropins	Amidated C-terminus Amidated C-terminus – Amidated C-terminus Amidated C-terminus – Amidated C-terminus – – Amidated C-terminus	Frogs Bees Frogs Frogs Frogs Toad Humans Bovine Frogs Insect	[29] [30] [31] [32] [33] [34] [35]
β-sheet peptides	Cathelicidins: -Protegrins -Bactenecin Defensins: -α-defensins -β-defensins -θ-defensins Tachyplesins Polyphemusin	Cysteine rich Disulfide forming loop/arginine rich Three disulfide bonds Three disulfide bonds Three disulfide bonds Cysteine/arginine rich Amidated C-terminus	Pigs Bovine Mammals Mammals Gorilla Horse crab Horse crab	[35] [36] [37] [38]
Extended/ flexible	Cathelicidins: -PR-39 -Tritrpticin -Indolicidin -Crotalicidin Histatines	Proline and arginine rich Tryptophan and arginine rich Tryptophan and amidated C-terminus Lysine rich Histidine rich and amidated C-terminus	Pigs Pigs Bovine Snakes Humans	[35] [39] [40]

Furthermore, one specific feature observed in all β-sheet peptides is being cysteine rich and full of disulfide bonds. These bonds increase the peptides stability as well as diminishing the effect of proteolytic enzymes on the peptide [25]. Β-sheet AMPs tend to keep a quite stable structure in both aqueous condition and membrane environment [26]. The well-known defensins comprise a large portion in this group and are produced by neutrophils, macrophages plus epithelial cells as inactive precursors [20, 22].

Ultimately, there are not many AMPs in nature to follow the extended/random coil formation. These AMPs have no secondary structure and are usually full of arginine, proline, tryptophan and/or histidine residues [19, 27]. Indolicidin is one of the best examples of this class, isolated from bovine neutrophils, with only 13 amino acid residues containing mainly tryptophan [28].

### Biochemical properties of AMPs

There are a number of principal features that are the same in nearly all kinds of AMPs regardless of their diversity in sources, structure and sequence. The first common key feature is hydrophobicity or the percentage of hydrophobic residues such as valine, leucine, isoleucine, alanine, methionine, phenylalanine, tyrosine and tryptophan in AMP sequence (typically 50%). Hydrophobicity is one of the essential factors that a cell membrane needs for its permeabilization. However, excessive hydrophobicity causes toxicity and loss of anti-microbial selectivity in mammalian cells [40, 41]. Furthermore, Chen et al. examined the effect of hydrophobicity of V13KL, a synthetic α-helical AMP, on hemolysis of human red blood cells (RBCs) and found that for a good anti-microbial performance,optimum hydrophobicity is required and any sequence with higher or lesser than that ideal level is likely to inactivate the peptide [41].

Amphipathicity is the next common property among AMPs and can be defined as the relative abundance of hydrophilic and hydrophobic residues or domains within the AMPs. In other words, it is the balance between the cationic and hydrophobic residues, in both the AMPs primary sequence and 2D/3D structure. Among all AMPs conformations, α-helix can show amphipathicity. It consists of peptides forming two faces of polar and non-polar which are actually hydrophobic and hydrophilic side chain of the residues [42].

Lastly, all AMPs show a net positive charge from + 2 to + 13 and might have a specific cationic domain. Lysine, arginine and sometimes histidine residues are said to be the reason for the AMPs cationic nature [43, 44]. It has been determined that an increase of charge from + 3 to + 5 in magainin 2 would enhance its antimicrobial effect against both gram-positive and negative bacteria. Meanwhile, an increase from + 3 to + 6 or + 7 results in more hemolysis as well as decreasing antimicrobial activity [44]. This last consequence is due to the powerful interaction between the peptide and the phospholipid head group that is likely to stop the peptide to enter the membrane [42].

## Antimicrobial activities of AMPs

Over the past few years, global public health has faced the emergence of multidrug-resistant microorganisms due to excessive use of antibiotics. This has created an urgent need for novel antibiotics to enter the clinical phases. In this regard, since the discovery of the magainins, the first AMPs discovered from the skin of the African clawed frog *Xenopus laevis* by Zasloff et al.*,* AMPs have become a potentially favorable future therapeutic candidate [45]. They are an important part of natural defense and immunity system to perform various effective mechanisms and thus kill the pathogens [46]. Cell membrane disruption, protein and DNA synthesis inhibition, suppressing vital cellular processes such as folding of proteins, synthesis of cell wall and metabolic turnover are a number of antimicrobial activities shown by AMPs [47].

### Mechanism of action:

In order to use AMPs for therapeutic purposes, we firstly need to know their mechanism of action (MOA). Earlier, it was assumed that the only function of AMPs is to disrupt the cell membrane resulting into cell death. However, today based on available evidences we know AMPs demonstrate a wide variety of mechanisms for microbial elimination [48]. Regardless of their structure, primary sequence or positive net charge, all of them have the ability to identify the microbial target. There are generally two classes for AMPs mechanism of action: (1) Direct killing, and (2) immune modulation [49].

#### Direct killing: membrane permeabilizing mechanism of action

There are basically two ways by which AMPs can target the cell membrane and disrupt it: (A) receptor-mediated (B) non-receptor mediated. Many of the bacterial AMPs such as nisin use the receptor-mediated way and these bacteriocins are active in vitro in the nanomolar range [50]. However, most AMPs produced by vertebrates and invertebrates tend to affect the cell membrane by manipulating its components and do not have any interaction with the receptors. These latter AMPs are typically active in vitro at micromolar levels [51].

**Fig. 1:**
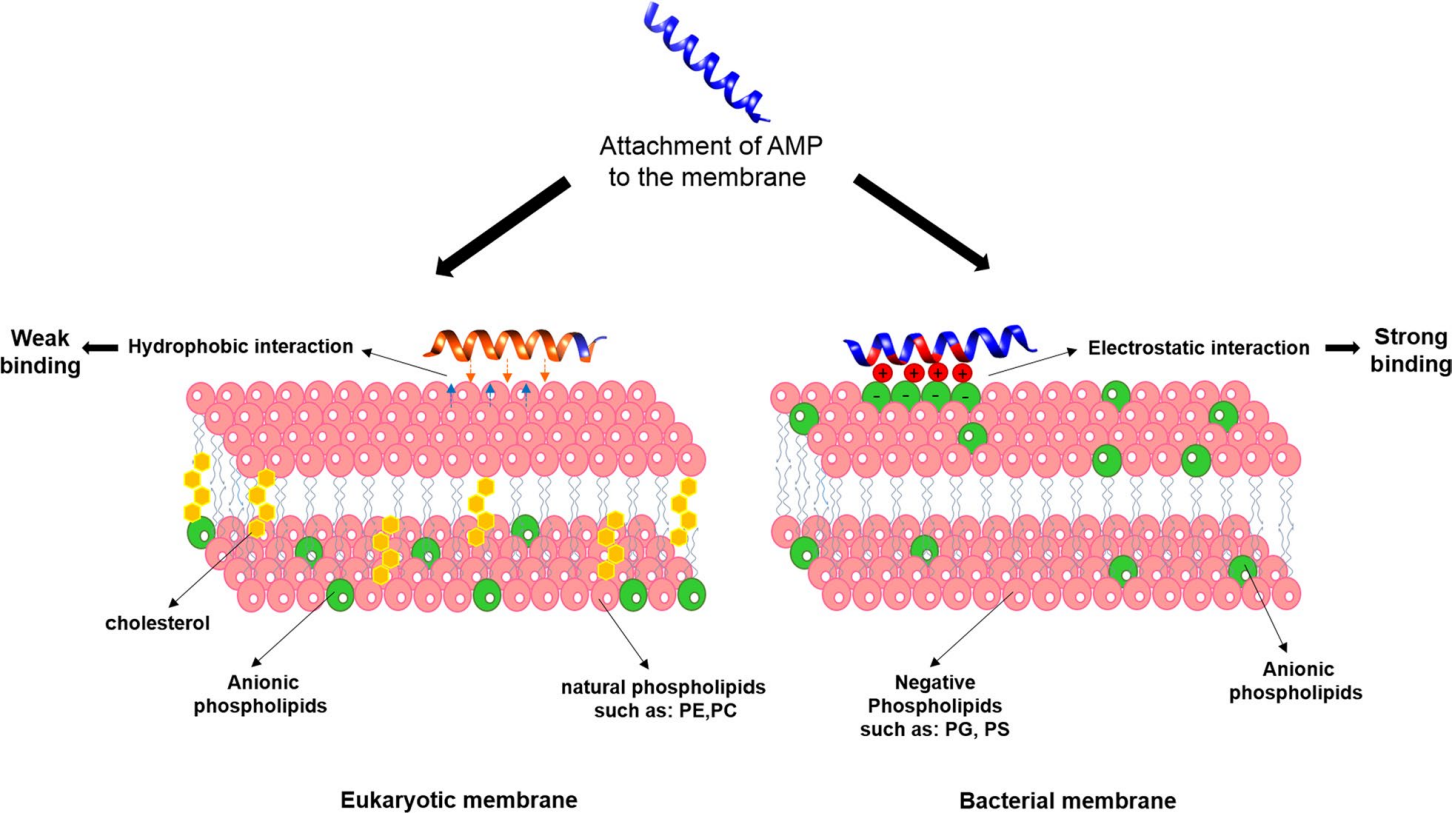
Interaction of cationic AMPs with the eukaryotic and bacterial membranes

Actually the outer cell envelope structure as the cytoplasmic membrane is the same in both gram-positive and gram-negative bacteria [52]. A thick peptidoglycan layer covers gram-positive bacteria, while gram negative bacteria have a thin peptidoglycan layer in addition to an extra outer membrane [2]. The reason why positively charged AMPs significantly attract bacterial membranes is due to membrane head groups with negative charge such as phospholipids, phosphtidylglycerol, cardiolipin, and phosphatidylserine. Moreover, the teichoic acid and LPS in gram-positive cell wall and gram-negative outer membrane respectively create an extra electronegativity to the bacterial surface [22, 53]. On the other hand, mammalian cell membrane is neutral in terms of net charge as it is filled with the zwitterionic phospholipid, phosphatydylethanolamine, phosphatydylcholine and sphingomyelin [53]. Moreover, there is asymmetric distribution of phospholipids in the mammalian cell membranes where zwitterionic phospholipids are localized in the outer leaflet and the negatively charged head groups, if available, in the cytoplasmic leaflet [22]., This is the reason why the interaction between mammalian cell membrane and AMPs is hydrophobic and undoubtedly weaker compared with AMP-bacterial membrane electrostatic interaction. Furthermore, mammalian cell membranes consist of cholesterol [2], which is supposed to stabilize the phospholipid bilayer and thus diminishing the AMPs activity [45]. It is also noteworthy to mention that bacterial negative transmembrane potential (-130 and -150 mV) is noticeably more than in mammalian cells (− 90 to − 110 mV) [54], influencing AMPs selectivity and, therefore, results in AMPs targeting on bacterial cells over the mammalian ones [42] (Fig. 1).

**Fig. 2:**
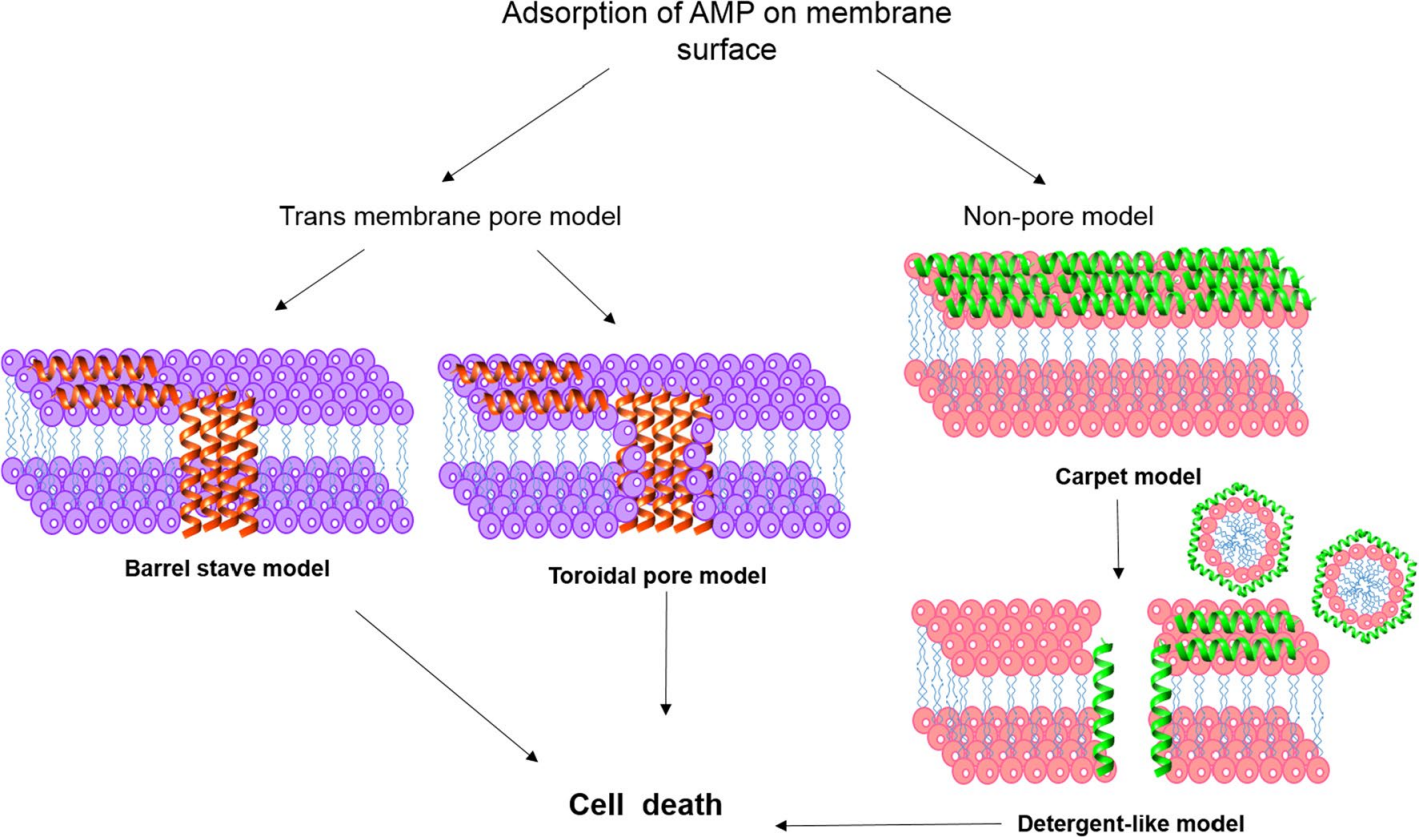
Mechanism of AMPs action on membrane permeabilization

There are a number of pore and non-pore formation mechanisms by which AMPs at optimum concentration are able to permeabilize the cytoplasmic membrane [52]. One considered model that is categorized under the transmembrane pore group is called ‘Barrel-Stave model’. In this model, AMPs are firstly placed alongside with the membrane and then enter into it vertically [55] (Fig. 2), building lateral peptide-peptide interactions, like membrane protein ion channels. The peptide amphipathicity plays a key role in creation of the pore as the hydrophobic region of the peptide align with the lipid region and hydrophilic region of the peptide contribute to the formation of the pore interior [21]. Not too many AMPs but some of them such as alamethicin [56], pardaxin [57] and protegrins [21] show this model for the killing of mammalian and bacterial cells.

Another explained model is known as ‘Toroidal pore model’ in which AMPs vertically enter into the lipid membrane but there is no peptide-peptide interaction formed [56]. However, the transient toroidal pores in this model are structured partially by both peptides and phospholipid head group (Fig. 2). The deeper AMP molecules are induced into the cytoplasmic membrane, the more lipid head groups are replaced and taken to the lipid tail area leading to the creation of toroidal pores, lipid disorder and change in membrane curvature [58]. As it is evident, the organization of hydrophobic and hydrophilic residues in bilayer membrane is manipulated and disrupted in toroidal model, while in barrel-stave model these arrangements remain same. Magainin 2 [26], lacticin Q [26], aurein 2.2 [59] and melittin [26, 56] demonstrated toroidal pores. Eventually, whether it is the AMPs forming barrel-stave or toroidal pore model, it all depolarizes the membrane and ultimately causes cell death.

On the other hand, some AMPs disrupt the cell membrane through ‘carpet mechanism’ which is also called as ‘detergent model’. In this model, an influential and critical concentration of AMPs must be adsorbed parallel to the lipid membrane and fully cover the cell surface forming the ‘carpet’ (Fig. 2). This creates a detergent-like model which eventually results in micelle formation and thus the membrane is disrupted leading to cell death. In this process, unlike the pore models, no peptide insertion into the membrane, peptide-peptide interaction and any particular peptide structures are formed [42]. Cecropin [60], indolicidin [61], aurein 1.2 [62], and LL-37 [51] are some of the AMPs to follow this mode of action. There are also other models of AMP action mechanism such as Shai-Huang-Matsazuki model, the interfacial activity model and the electroporation model [26].

**Fig. 3:**
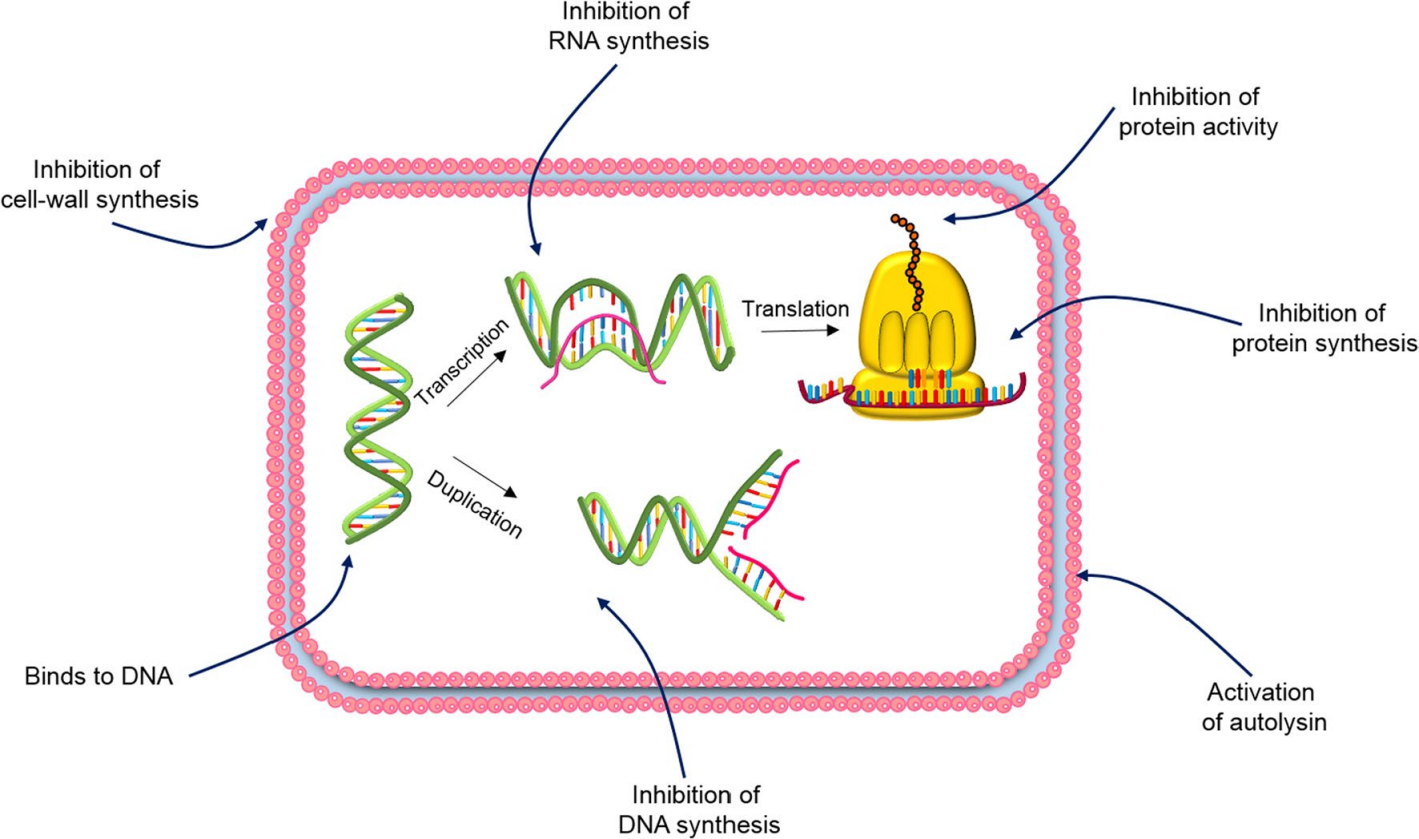
Non membrane targeting mechanism of action of AMPs

Not all AMPs target cell membrane disruption to kill the cell; some of them target other parts, including the cell wall or intracellular targets, in bacteria (Fig. 3). In order for AMPs to kill the cell by affecting intracellular components, they initially have to interact with the cytoplasmic membrane to pass through. AMPs with this approach usually tend to influence vital processes such as inhibiting the synthesis of proteins/DNA and inhibiting the protein/enzymatic activities [21]. Buforin II, a histone derived AMP found in frogs, is the example of an AMP that passes through *E-coli* membrane causing no damage to it, and ultimately binds to the bacterial DNA and RNA [1]. Human α defensin 5 also exerts its antimicrobial effects by entering *E-coli* and accumulating at the cell's opposite poles and division plate. Indolicidin [1], human β-defensin 4 [63], human α-defensin 1 and PR-39 [1] are some other AMPs to destroy the cell by attacking intracellular targets.

#### Immune modulation mechanism of action

Interestingly, some AMPs activate and employ immune cells, leading to an improved response for microbial killing and/or inflammation control [64]. Hence, AMPs present in neutrophils and macrophages are essential components of innate immune system as the first line of defense against pathogens [16]. When an infection occurs, immune responses are generated to attract immune cells at the infection site and inflammation is controlled. Activation, attraction, and differentiation of white blood cells, stimulation of angiogenesis and reactive oxygen/nitrogen species are different kinds of immune responses generated by AMPs [65]. Additionally, exaggerated and damaging pro-inflammatory responses like sepsis are avoided by other immunomodulatory activities of AMPs such as suppression of toll-like receptors (TLR) and/or cytokine-mediated production of inflammatory cytokines and anti-endotoxin activity [66]. LL-37 and β defensins are human chemoattractive AMPs to command mast cells [67], leukocytes [68] and dendritic cells to the infection site [69]. Furthermore, there are synthetic versions of AMPs available called as innate defense regulators (IDR) that play a role in suppressing pro-inflammatory cytokines when mice are infected (IDR-1 and IDR-1018) [70]. Mice severely infected by malaria were given anti-malaria drugs plus IDR-1018. A significant reduction occurred in the neural inflammation that would normally cause death in the infected mice, suggesting that IDR-1018 is indirectly responsible for this inflammation control. Moreover, there is also proof showing that AMPs not only participate in innate immune system but also affect adaptive immune system, the T and B cells, although it is yet to fully be examined and understood [64]. There are number of models indicating the AMPs immunomodulatory mechanism in mammalian cells [22]: ‘Alternate ligand model’ suggests that AMPs directly bind to the particular cell membrane receptors for downstream signaling cascades. Meanwhile, in ‘membrane disruption model’, the AMPs indirectly affect the receptor activation by altering a specific site of membrane that contains the receptor. In another model called ‘transactivation’, a membrane-bound factor is released because of the AMPs effect, which could bind to its receptor afterwards. Finally, AMPs are also able to prevent inflammation by collecting and clearing the endotoxin LPS, which normally binds to the TLR4 causing inflammation [22].

## Anticancer activities

Cancer is a leading cause of death worldwide. It arises from the transformation of normal cells into tumor cells that grow beyond their usual boundaries, turning into tumor masses. They possess angiogenesis enabling it to spread and invade other parts of the body (metastasis) [76]. Lung cancer is the most common while colorectal cancer is the second one followed by prostate and breasts cancers [7]. In case of cancer treatment, doctors often recommend chemotherapy which is an aggressive form of chemical drug therapy meant to destroy rapidly growing cells [77]. However, there are several unfavorable side effects of chemotherapy such as multiple drug resistance [78] and the lack of drug selectivity [78, 79]. Therefore, currently, antineoplastic agents of higher selectivity with lesser side effects are in great demand [80, 81]. Therapeutic peptides are acknowledged as a new potential and favorable option for cancer therapy [82]. Boohaker et al*.* [83] classified therapeutic anticancer peptides into three general groups- (A) anti-microbial/pore-forming peptides that are naturally produced by all living creatures, also known as anticancer peptides or ACP, (B) cell-permeable peptides and (C) tumor targeting peptides [84]. Therapeutic peptides have many significant advantages such as their small size, high activity, specificity and affinity, least drug-drug interaction, ability to pass through the membrane and no sign of AMP accumulation in vital organs like kidney and liver decreasing the toxic side effects (Table 2) [82]. Moreover, being easily synthesized and modified [83] as well as being less immunogenic than recombinant antibodies and proteins are other beneficial features of AMPs [85].

**Table 2:** AMPs as therapeutic agents

Peptide	Phase	Application	Sources	Route of administration	References
Iseganan	III	Oral mucositis in patients receiving radiotherapy for head and neck malignancy	Ptotegrin-1 (pigs)	Oral solution	[71, 72]
TD-1792	III	Gram positive infections/ skin and soft tissue infections	Synthetic peptide	Topical	[73]
CZEN-002	IIb	Vaginal candidiasis	αMSH (human)	Vaginal gel	[73]
NP-432	Pre-clinical	Methicillin-resistant *Staphylococcus aureus* (MRSA) / *P. aeruginosa C*. *difficile* infections	Synthetic peptide	Intravenous	[73]
lytixar	I/II	Uncomplicated gram-positive skin infections, impetigo, nasal colonization with *S. aureus*	Synthetic antimicrobial peptidomimetics	Topical hydrogel	[74]
C16G2	II	Dental caries	synthetic	Topical	[73]
Omiganan	II/III	Catheter infection and rosacea	Indolicidine (bovine)	Topical gel	[75]
TD-6424	III	Osteomyelitis Bacterial infection	Synthetic peptide	Intravenous	[73]
PXL01	II	Prevention of post-surgical adhesion formation in hand surgery	Lactoferricin (human)	Hyaluronic acid based- hydrogel for administration at the surgical site	[2]
hLF1-11	I/II	Bacteremia and mycosis in immunocompromized haematopoetic stem cell transplant recipients	Lactoferricin (human)	Intravenous treatment	[75]
Novexatin	II	Onychomycosis	Defensins (human	Topical brush-on treatment	[75]
LL-37	I/II	Hard-to-heal venous leg ulcers	LL-37 (human)	Solution for administration in the wound bed	[75]
PAC-113	II	Oral candidiasis in HIV seropositive patients	Histatin3 (human saliva)	Mouth rinse	[75]

### Mechanism of ACP action:

There are practically two mechanisms by which ACPs affect the membrane and cause cell death: necrosis and apoptosis.

A normal cell holds 3–9% phosphatidyl serine (PS) of the total amount of phospholipids in its inner-leaflet making it neutral [86]. Cancer cell membranes typically have a high negative net charge as they hold PS in their outer leaflet [87]. Moreover, heparin sulfates and O-glycosylated mucins on the tumor cells surface [88], highly negative potential of the cell, elevated membrane fluidity and surface area [89], altogether create a great electrostatic interaction between anionic membrane and the cationic ACPs [90]. It has been proved that in the membranes of cancer cells, as in leukemia and lung cancer, there is lesser content of cholesterol [91]. Consequently, the membrane fluidity is increased and it becomes destabilized, enhancing the lytic activity of ACPs such as cecropins [92]. However, the role of cholesterol in activating ACPs is still uncertain. Finally, cancer cells tend to have a greater surface area than normal cells as they transform and possess a lot of microvilli. This allows for an increased number of ACPs to bind to the cancer cells. After being attached to these cells, AMPs tend to destroy them through necrosis or apoptosis [6]. Overall, changes in the cancerous cell membrane contents and morphology are cancer biomarkers to be identified by ACPs (Table 3).

**Table 3:** Therapeutic peptides and their uses in cancer therapy

Peptide	Source	Mechanism	References
Lactoferricin B	Bovine	Apoptosis	[93]
SALF	Shrimp	Apoptosis	[94]
KLA repeat AMP [(KLAKLAK)2]	Synthetic	Apoptosis	[95]
Pardaxin	Fish	Apoptosis	[96]
Tat-bim	Fusion of Tat and Bim peptides	Apoptosis	[97]
Poropeptide-Bax	Bax	Apoptosis	[98]
R8-Bax	Fusion of poropeptide-Bax with argenine	Induced cell death	[98]
CT20-NP	Derived from Bax	Interruption the membrane integrity	[99]
RRM-MV	Synthetic peptide	cytotoxic to different cancerous cell lines	[100]
TIP	Derived from p53	Inhibition of p53-MDM2 interaction	[101]
PNC-27	Synthetic peptide	necrosis	[102]
Kahalalide F	Marine-derived peptide	necrosis	[103]
Polybia- MPI	Natural ACP	Induction of necrosis in various leukemia cells	[104–106]
ABT-510	De novo design	Inhibition of tumor angiogenesis	[107, 108]
HNP-1	Human	Inhibition of angiogenesis	[104, 109]

#### Induction of tumor apoptosis

There are two pathways in which a cell dies through: apoptosis and necrosis. Apoptosis is a highly regulated process of programmed cell death for the elimination of unwanted cells, helping the cell population remain stable in tissues [110]. Furthermore, cells undergoing development or cellular stress might be damaged beyond repair and here,too, apoptosis plays a vital role [111, 112]. If apoptosis is for some reason stopped or prevented, it can lead to uncontrolled cell division and subsequently development of a tumor, metastasis and resistance to cancer therapeutics [113].

**Fig. 4:**
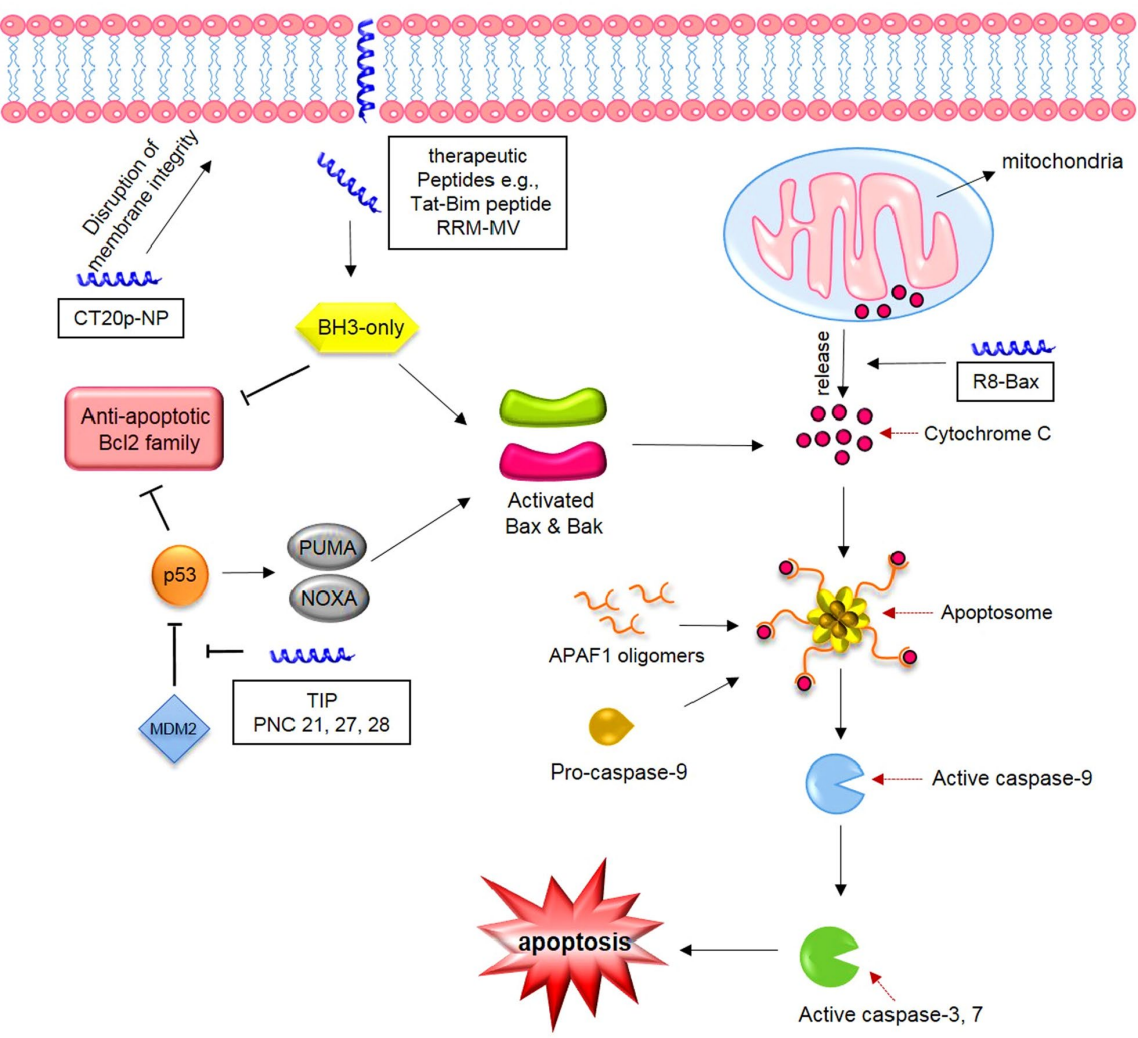
Therapeutic peptides and their roles in apoptosis

There are mainly two pathways for apoptosis initiation: intrinsic and extrinsic. Intrinsic pathway is under control of Bcl-2 family protein members (e.g., Bcl-2 and Bcl-XL) (promoting cell survival) and pro-apoptotic proteins (e.g., Bax and Bak) (promoting cell death) [114] and [115]. When a cell is stressed, apoptotic signals are generated meaning that the cell is infected or the DNA is being damaged. Throughout these signals, BH3-only proteins activate pro-apoptotic proteins, namely Bak and Bax which are in charge of cell death either by directly binding to them or by inhibiting anti-apoptotic proteins such as Bcl2 and Bcl-XL which indirectly results in Bak and Bax activation. This eventually leads to the formation of pores in mitochondrial outer membrane [116] and cytochrome c is then released into the cytosol. Cytochrome c subsequently activates apoptotic protease-activating factor-1(APAG-1) and procaspase-9 by binding to them and as a result apoptosome is created [117]. Finally, the apoptosome influences and activates caspase-9 which itself activates procaspase-3 and -7 leading to apoptosis [118] (Fig. 4). Overexpression of anti-apoptotic proteins interrupts apoptosis [113] and thus onset of cancers such as prostate, neuroblastoma, kidney, breast cancer, acute lymphoblastic leukemia, chronic lymphoblastic leukemia and non-Hodgkin’s lymphomas happens. Generally, it is such a good idea to examine and target apoptosis pathways for effective use of therapeutics in premalignant and malignant cells [119].

#### Target tumor suppressor proteins

While p53 level in normal and healthy cells is low due to its rapid degradation by ubiquitin-dependent proteolysis [120]but it increases in damaged cells [121], leading to apoptosis. However, the activity of p53 is inhibited in many cancers through overexpression of MDM2, which acts as p53 repressor protein that binds to p53 and limits this transcription factor and is able to cause its quick degradation. In order to stop the rapid degradation of p53 by obstructing the interaction between MDM2 and p53, several peptides were designed from p53 amino acid sequence [122]. Bottger et al. separated TIP peptide from the N-terminal MDM2- binding domain region of p53 blocks p53-MDM2 interaction, results in increased levels of p53 in addition to its activation as a transcription factor [101].

#### Induction of tumor necrosis

Necrosis is another form of cell death caused by external factors. Many accidental (physical or chemical injury) or pathological conditions lead to unregulated digestion of cell components. Chromatin flocculation, swelling, degeneration of the cytoplasm and the mitochondrial matrix, cellular blebs, spilling of the cytoplasmic contents into the extracellular space occur in necrosis [123]. Necrosis inducing peptides are considered to be an exceptionally novel class of anticancer agents since these peptides have the ability to disrupt the membrane by their lytic activity, have better selectivity than traditional chemotherapeutic drugs in addition to preventing multidrug resistance [124].

#### Inhibition of tumor angiogenesis

Angiogenesis is one of the fundamental steps in transition of a benign to malignant tumor. It is a process through which new blood vessels are formed from pre-existing ones. Although it is a normal and essential process in a healthy body, in cancer it provides vital nutrients and oxygen for the tumor as well as carrying away its wastes [125]. Tumor angiogenesis occurs under the influence of fibroblast growth factor (FGF), epidermal growth factor (EGF), vascular endothelial growth factor (VEGF), placental growth factor (PLGF),tumor necrosis factor-alpha (TNFa), platelet-derived growth factor (PDGF) and angiogenin (Ang) [126, 127]. However, today many peptides are known to have anti-angiogenesis and antitumor effects by blocking the interaction between growth factor and its receptor on the cancerous cells.

#### Immunomodulatory function

**Fig. 5:**
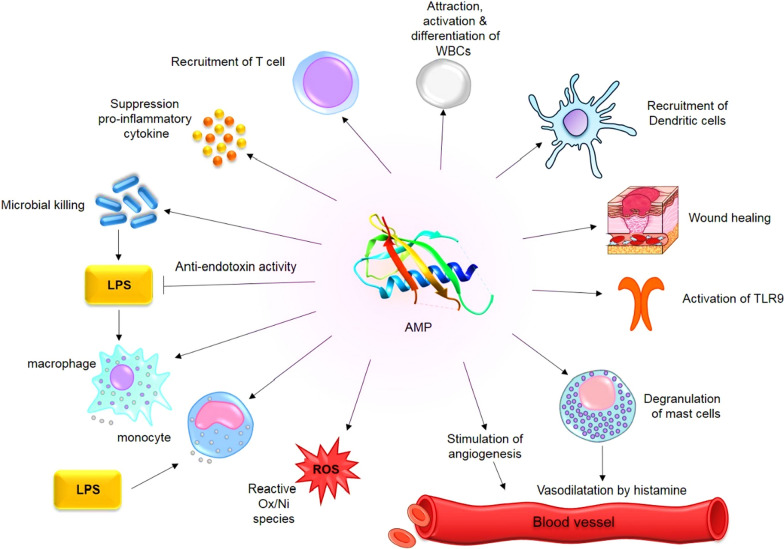
Immunomodulatory mechanism of action of AMPs

Malignant tumors tend to produce cytokines and growth factors affecting a wide range of cells like endothelial cells, inflammatory immune cells and fibroblasts. These tumor controlled cells and the molecules of extracellular matrix together are considered as the tumor microenvironment [128] where antitumor immunity and tumor-originated pro-inflammatory activity are finely balanced and disadvantaging the antitumor immunity [129]. There are a number of bioactive peptides that have been proved to be effective in treatment of cancer by modulating the immune responses (Fig. 5) [130].

## AMPs as therapeutic agents; challenges and improvements

### Challenges

Although the advantages of utilizing AMPs are widely proved due to their antimicrobial and anticancer effects, only a few of them are going through clinical trials and developments [131]. Evidently, scientists are facing several technical, regulatory, and commercial challenges including AMP’s susceptibility to proteolysis, their poor pharmacokinetic (PK) properties and high cost of production.

There are a number of ways available to improve AMPs' PK features. Firstly, AMPs stability could be enhanced by employing D-enantiomers [132]. Furthermore, cyclization of AMPs by joining the backbone N- and C-terminals or by disulfide bridges similar to human defensins [133] improves their serum stability and takes place. End-tagging by hydrophobic oligo amino acid stretches [134], and blocking or exerting changes on the N-or C-terminals of AMPs including N-acetylation, N-pyroglutamate or C-amidation are other approaches for increasing the AMPs resistance against proteolytic enzymes [135]. Another approach is PEGylation or the addition of polyethylene glycol (PEG) to AMPs [136, 137] resulting in lesser host toxicity [138]. In fact, PEGylation aids improving AMPs PK properties [139] by increasing AMPs hydrophilicity. Thus PEG plays the role of a protective shield for AMPs protease digestion, extends their circulation time as well as decreasing of glomerular filtration rate [140, 141]. Tachyplesin I, magainin 2 [142] and nisin undergone PEGylation being benefited with the mentioned features [143].Targeted delivery of the peptide to a cancer cell is enabled through a strategy called as liposomal delivery in which the lysosome is tagged by a cancer cell-specific ligand [144]. On the other hand AMPs could also be designed as a component of nanoparticles that would help enhance their PK properties but this is yet to be fully examined [145]. Moreover, the amino acid substitution may improve the therapeutic function of peptides. For example, the EGFR lytic-peptide potentially leaves cytotoxic effects on various groups of cancer cells which are resistant to anti-EGFR antibodies and EGFR tyrosine kinase inhibitors (TKIs) [146]. In order to enhance the anticancer activity of this peptide, arginine could substitute for the second histidine in EGFR lytic-peptide, leading to create a new bioactive form known as EGFR(2R)-lytic with better binding affinity for sticking to the EGFR of cancer cells and stronger anticancer activity than the unmodified version [147].

Overall, therapeutic peptides have low metabolic stability, the main reason why they are less likely to be applied in clinical trials. In case of oral administration, there is the possibility of proteolytic digestion of AMPs by trypsin and pepsin present in the digestive system [1]. On the other hand, systemic administration of the AMPs, turned out the possibility of AMP degradation by proteolytic enzymes, dropping off the AMPs half-lives while organs like liver and kidneys quickly eliminate them from the circulatory system [148]. Hence, local application of AMPs seems to be the best and most logical choice, although there is a still the possibility of AMPs being decomposed by tissue proteases. Applying dermal creams and emollients to the skin, wounds or surgery site plus nasal spray for mucosal delivery are some of the examples of AMPs local applications.

Another challenge involved with AMPs is the significant overpricing of synthesis and development of these peptides. Solid phase peptide synthesis with 50 amino acid residues at most [2] is an available and frequently used chemical method for the synthesis of these therapeutic peptides [149]. Other production methods of recombinant peptides by using bacteria, yeast, insect and mammalian cells proved to be efficient and cost effective.

### Strategies to improve AMPs

AMPs isolated directly from natural sources are not adequately well-rounded for therapeutics. However, they have the potential to gain better efficacy, safety and stability after undergoing a number of strategies like recombinant technology, innovative formulation and design of drug delivery systems. Unfortunately, these crucial fields have received the least attention. For example, in formulation strategy, therapeutic peptides with minus side effects and an improved efficacy are resulted as this method provides the possibility to target the delivery of AMPs to a particular tissue while the drug release over time is also controlled [150].

#### Recombinant technology

Chemical synthesis of peptides is a complex and costly process with little output in the end [151], while genetic engineering is more of an efficient method which produces AMPs [151] in a larger scale by using various microorganisms including bacteria and yeasts as host cells [152]. For example, *Pichia pastoris* is a methylotrophic yeast commonly exploited for recombinant productions [153]. *P. pastoris* is responsive to high cell density fermentations and only requires an inexpensive medium to quickly grow in [154]. It is a non-pathogenic microorganism able to directly secret useful proteins and peptides into the culture medium [155]. The fowlicidin-2 is a recombinant peptide produced by *P. pastoris* X-33 with the expression vector pPICZa-A, displaying a wide range of antimicrobial, hemolytic and anticancer activities [156]. However these recombinant AMPs are susceptible to yeast and bacterial proteases plus that exerting adequate post translational modifications on them, such as disulfide bond, is generally impossible [157]. Furthermore, plants are also considered as a good alternative candidate for AMPs production [158]. Since they are able to finely do the glycosylation, folding and disulfide bond formation in recombinant AMPs necessary for their biological activities [159]. The leaves in plants such as lettuce, alfalfa, clover and tobacco, more specifically, are appropriate platforms for lasting expression of recombinant proteins [160]. LF chimera is a peptide with a wide spectrum of antimicrobial activity which is originally made of the combination of another two anti-microbial peptides, namely lactoferricin (17–30) and lactoferrampin (265–284) connected at their C-termini. Interestingly, LF chimera has a shorter incubation time and demonstrates its antimicrobial activity in a lower required concentration than the two peptides it is made of [161]. LF chimera is an example of a recombinant peptide that can be produced by tobacco when its sequence is placed in the plant and fused with endoplasmic reticulum retention signals along with CaMV 35S promoter and then transferred by agrobacterium-mediated transformation [162]. The bacterial recombinant system used for heterologous protein expression was *Escherichia coli* BL21 (DE3). JAMF1, a recombinant AMP produced in bacterial systems. This peptide consists of Human α-defensin 5, Secretory phospholipase A2 (sPLA2) and gelsolin (an actin-binding protein). The efficacy of JAMF1 has been proved against both gram-negative and gram-positive bacteria like *E. coli* DH5α, extended-spectrum beta-lactam-resistant *Enterococcus* spp. (SHV-12 & CTX-M-14),), carbapenem-resistant *Klebsiella pneumoniae* (KPC) and quinolone-resistant *K. pneumoniae* (qnrA) [163].

#### Nanobiotechnology

Nanotechnology is one of the strategies through which AMPs become significantly more efficient and their unfavorable natural or synthetic features are amended [164]. The main role in this method is played by nanocarriers. a suitable choice for drug delivery process. It enables formulation design for specific tissue delivery, controlled release of the drug over time due to controlled carrier degradation while the metabolic and chemical stability of the AMPs is quite maintained [165, 166]. The nanocarriers are usually made of biocompatible and biodegradable materials such as lipids (e.g., phospholipids, triglycerides, cholesterol and monoolein) and polymers (e.g., Cellulose, chitisan, hyaloronicacid, polylactic-co- glycolicacid (PLGA) and polylacticacid (PLA). Hyaluronicacid nanogels exemplify one of the successful nanocarriers for LL-37 analog LLKKK18, improving the AMPs anti-microbial activity against mycobacteria both in vivo and in vitro [167]. The mentioned nanocarrier not only increases the peptides resistance for proteases but also eliminates the toxicity against the host cell [167]. Moreover, liquid crystalline lipid nanoparticles and lipid nanocapsules have also shown to be a potent candidate for carrying some AMPs possessing various biophysical features. The AMPs characteristics like efficacy and antimicrobial functions were enhanced when carried by these nanoparticles [168]. Overall, although nanoformulations as delivery system for AMPs have been examined on laboratory animals, there is a promising approach for this method to be brought at clinical level.

##### Mechanism of actions of nanocarriers

Nanocarriers can transport their containing substance toward the target in two principal ways: passive and active targeting. In passive targeting or non-directive delivery, there is no surface modification but the nanocarrier's size and shape is controlled [169]. In active targeting or directed delivery, the nanocarrier's surface is decorated by ligands binding to tumor cell receptors and this increases the nanocarrier affinity for cancer cells, thus enhances the quantity of the drug delivered to a specific tissue. Comparing both systems, it is evident that the former delivery system usually has fewer agents and consequently is easier to be prepared as compared with the later one. However, as the active targeting is facilitated with ligands, it creates a better interaction with the drug delivery system and the targeted site [170].

##### Conjugation with gold nanoparticles

 HP (2–20) is a peptide with 19 amino acids that has great antimicrobial activity against bacteria, fungi, and protozoa with no sign of hemolysis and is separated from the N-terminus of *Helicobacter pylori* ribosomal protein L1 (RpL1). On the other hand, HPA3P is a peptide taken from HP (2–20) that has undergone several amino acid substitutions [171]. A new delivery system that is being considered these days is by taking advantage of gold nanoparticle-DNA aptamer (AuNP-Apt) conjugate [172] which has shown little toxicity and no immunogenicity, so far [173]. It has been proved that conjugating AMPs with gold as the nanocarrier makes the drug delivery process much more effective in mice [174]. In the study, there were two groups of mice infected with *Vibrio vulninficus,* a gram-negative, highly virulent bacterium that causes gastroenteritis, primary sepsis, and wound infection in humans. The result showed that mice in control group had died of infection while mice treated with AuNP-AptHis-HPA3P^His^ had survived. Furthermore, it was clear that treating the mice with HPA3P^His^ alone, also resulted in their death. These outcomes suggest that conjugation of HPA3P^His^ to the gold nanoparticle, AuNP-Apt^His^, is significantly beneficial. As it improves the peptides stability against proteolytic enzymes, makes the delivery process efficient and no host toxicity. Furthermore, it is a simple system to work with since AuNP-Apt^His^ conjugates can stick to any kind of AMP which had been previously tagged with His. Besides, a single administration is effective enough as these gold particles provide a long-lasting efficacy. Nevertheless, more preclinical research has to be done to confirm the safety of this specific method and to determine whether it is an economically affordable process to step up for a large-scale production [172].

##### Challenges and limitations of AMPs and Nanotechnology

Till date, although plenty of AMPs (> 3000) have been discovered and characterized, almost all of them have disappointed scientists who hoped to use them for human medication purposes. However, there are still a few AMPs that have successfully passed clinical trials and received FDA approval to enter the market, namely gramicidin D, daptomycin, vancomycin, oritavancin, dalbavancin, and telavancin [175, 176].

It is interesting how there is no synthetic AMP approved by FDA [177]. Production of therapeutic peptides is expensive enough and using nanocarriers for drug delivery system would add extra cost on to it. Possibility of the peptides being exposed to natural solvent through the process of preparing nanodelivery systems is another potential challenge. Furthermore, the drugs are likely to interact with the wall of nanocarriers leading to incomplete release and poor bioavailability of the drugs [178].

## Conclusion

Infectious diseases and cancers are the two health challenges globally. Demonstrating different mechanisms of action, AMPs are the new promising and potential therapeutic candidates for these two lethal concerns. Furthermore, various methods and strategies have been considered and applied to improve different features of AMPs like modifying the existing AMPs, synthetizing new ones as well as preparing and employing various delivery systems for these peptides. Overall, there is a chance for these novel candidates to be confirmed in terms of their therapeutic benefits and hopefully lead to market authorization of several new AMP-based drugs.

## Data Availability

Not applicable.

## Abbreviations

AMP: Antimicrobial peptide
ACP: Anticancer peptide
MOA: Mechanism of action
MIC: Minimum inhibitory concentration
hCAP18: 18-KDa human cathelicidin antimicrobial protein
LPS: Lipopolysaccharide
TLR: Toll-like receptor
ROS: Reactive oxygen species
PLA: Polylacticacid
GFR: Gelomerular filteration rate
FGF: Fibroblast growth factor
EGF: Epidermal growth factor
VEGF: Vascular endothelial growth factor
PLGF: Placental growth factor
TNFa: Tumor necrosis factor-alpha
PDGF: Platelet-derived growth factor
Ang: Angiogenin
IDR: Innate defense regulators
PK: Pharmacokinetic
